# PUMA-mediated epithelial cell apoptosis promotes *Helicobacter pylori* infection-mediated gastritis

**DOI:** 10.1038/s41419-020-2339-x

**Published:** 2020-02-20

**Authors:** Yini Dang, Yifeng Zhang, Lingyan Xu, Xiaoying Zhou, Yanhong Gu, Jian Yu, Shidai Jin, Haoming Ji, Yongqian Shu, Guoxin Zhang, Shiyun Cui, Jing Sun

**Affiliations:** 1 Department of Gastroenterology, The First Affiliated Hospital of Nanjing Medical University, Nanjing, 210029 China; 2 Department of Gastroenterology, Nanjing First Hospital, Nanjing Medical University, Nanjing, 210001 China; 3 Department of Oncology, The First Affiliated Hospital of Nanjing Medical University, Nanjing, 210029 China; 4 Department of Pathology and Radiation Oncology, University of Pittsburgh School of Medicine, Pittsburgh, PA 15213 USA; 5 Department of Oncology, Haian People’s Hospital, Nantong, 226630 China

**Keywords:** Chronic inflammation, Gastroenteritis

## Abstract

The molecular mechanism responsible for *Helicobacter pylori* infection-mediated gastritis and carcinogenesis is not yet clear. Increased evidence suggests that chronic gastritis and elevated gastric epithelial cell (GEC) apoptosis are crucial events during stomach carcinoma transformation. PUMA is a potent proapoptotic Bcl-2 protein and mediates acute tissue injury. In this study, we aimed to investigate the role of PUMA in GEC apoptosis and inflammation induced by *H. pylori* infection. As a result, we found that PUMA expression was elevated in gastritis tissues compared with uninvolved tissues, and it was correlated with the severity of apoptosis and gastritis. In mice, *PUMA* mRNA and protein were markedly induced in GECs upon induction of gastritis by *H. pylori*. *PUMA*-deficient mice were highly resistant to apoptosis and gastritis induced by *H. pylori*. Furthermore, the transcription factor NF-κB p65 binds to *PUMA* promoter to activate *PUMA* transcription after *H. pylori* infection. In addition, NF-κB inhibitor could rescue *H. pylori*-induced apoptosis and gastritis. Finally, *H. pylori*-induced activation of *p-p65* and *PUMA* was mediated via Toll-like receptor 2 (TLR2) and blocked in *TLR2* knockout mice. Taken together, these results verified the pro-inflammatory effect of PUMA in *H. pylori-*infected gastric tissue. Moreover, TLR2/NF-κB-mediated transcriptional regulation of *PUMA* contributes to the pathogenesis of *H. pylori*-infected gastritis.

## Introduction

Inflammation is considered a hallmark of cancer, and chronic inflammation plays an essential role in the development of several types of solid tumors. Infection with *Helicobacter pylori* and the resulting chronic inflammation might be the initial step in stomach carcinogenesis. Once acquired, infection can persist and lead to elevated gastric epithelial cell (GEC) apoptosis and chronic gastritis that can progress to gastric atrophy, metaplasia, and finally gastric carcinoma^1,2^.

Inflammatory responses induced by *H. pylori* infection play a pivotal role in human chronic gastritis^3–5^, partly by activating a complex network of immune signaling, including the nuclear factor (NF)-κB pathway. Cytotoxin-associated gene A (CagA), lipopolysaccharide (LPS), and peptidoglycan are known virulence factors that significantly contribute to *H. pylori*-induced activation of NF-κB and its target genes^6,7^ as well as chronic inflammation^4,6,8–10^. However, the key downstream NF-κB-dependent targets in GEC apoptosis and chronic inflammatory responses remain to be identified in *H. pylori* infection-mediated carcinogenesis.

p53 upregulated modulator of apoptosis (PUMA) is a BH3-only Bcl-2 family member^11,12^ and functions as a critical initiator of apoptosis in p53-dependent and -independent manner^13^. PUMA potently induces mitochondrial permeabilization, cytochrome C release, and apoptosis by binding to other Bcl-2 family members, such as Bax, Bcl-2, and Bcl-X_L_^14–16^. We have previously reported that PUMA is directly activated by p65 through the canonical NF-κB pathway during colonic inflammation in both humans and mice^17^, and it mediates inflammation as well as tumor necrosis factor (TNF)-α-induced intestinal epithelial cell apoptosis^18,19^, suggesting a potential role of PUMA in gastrointestinal inflammation and tissue injury.

We hypothesized that PUMA might be involved in the pathogenesis of gastric cancer by mediating GEC apoptosis induced by *H. pylori* and contribute to chronic gastritis. In this study, we found that PUMA is induced by *H. pylori* by Toll-like receptor 2 (TLR2)/NF-κB-mediated transcriptional regulation and contributes to GEC apoptosis, gastritis, and the progression of gastric cancer, which is significantly attenuated by genetic ablation of PUMA or TLR2.

## Results

### Elevated PUMA expression and apoptosis in *H. pylori*-positive human gastric tissues

**Fig. 1:**
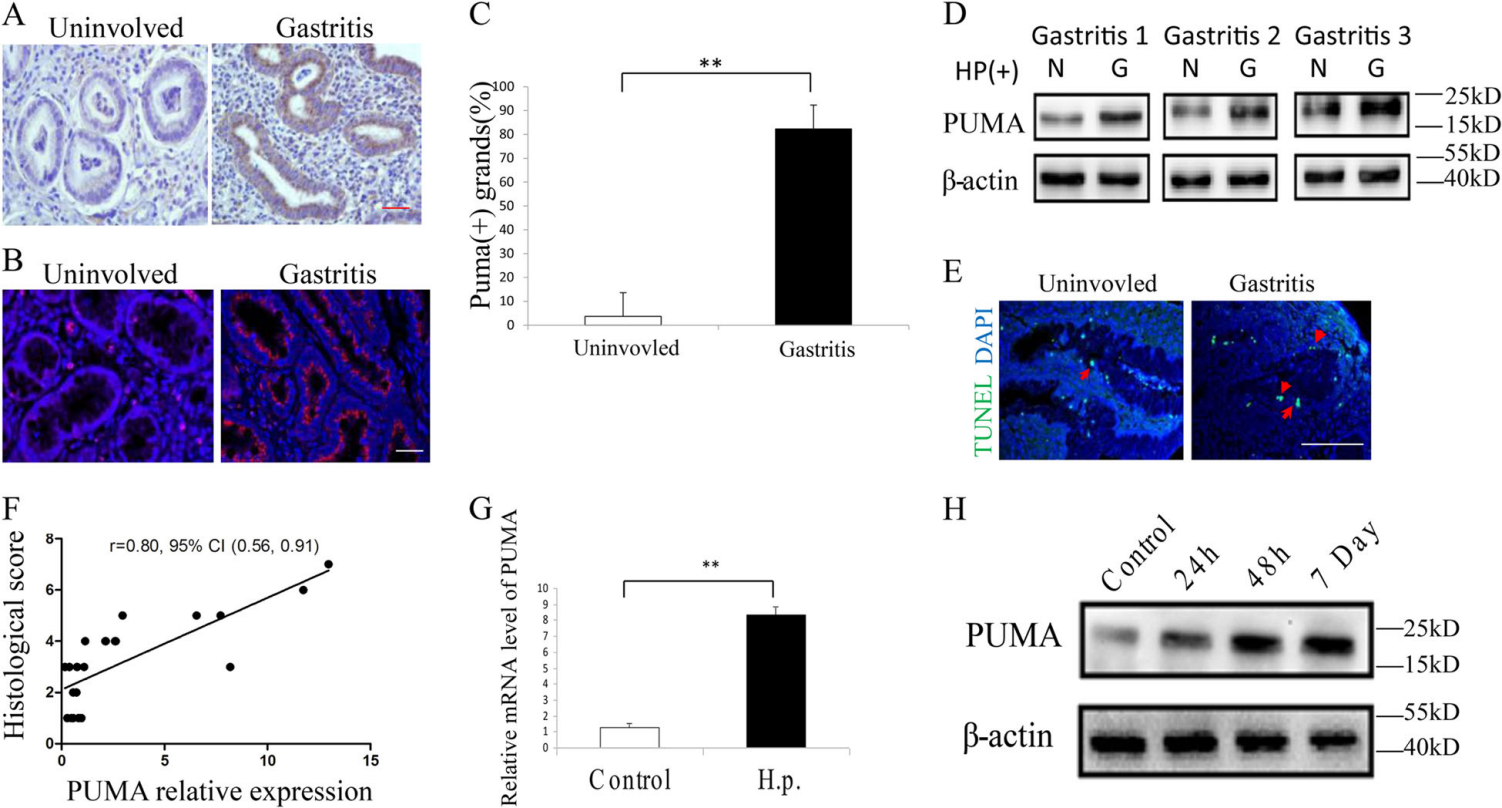
Apoptosis and PUMA induction in *H. pylori*-positive human gastritis specimens. **a**, **b** IHC (**a**) and IF (**b**) staining of PUMA in a matched pair of uninvolved gastric and gastritis tissues (×400), including 20 matched *H. pylori*-positive pairs of human gastric tissues with uninvolved or different stages of gastric mucosal diseases. **c** The results of the statistical analysis are presented as the mean ± SEM. **P < 0.01. **d** Western blot analysis of *PUMA* expression in three matched pairs of uninvolved gastric (N) and gastritis (G) tissues. **e** TUNEL staining of a matched pair of uninvolved gastric and gastritis tissues. **f** Correlation between PUMA protein expression and histological score in gastritis patients, N = 20. **g** RT-PCR analysis of *PUMA* expression revealed increased induction of PUMA in *H. pylori*-positive gastric cancer cells compared with the negative controls (*N* = 4/group). The results were presented as the mean ± SEM. **P < 0.01. **h** Western blot analysis was performed at 24 h, 48 h, and 7 days after *H. pylori* treatment in mice (*N* = 4/group).

To study the role of PUMA in *H. pylori* infection-mediated carcinogenesis, we first analyzed 20 pairs of matched *H. pylori*-positive human gastritis tissues with uninvolved tissues. *PUMA* was found to be elevated in the gastritis tissues compared with uninvolved tissues using immunohistochemistry (IHC) and immunofluorescence (IF) staining (Fig. 1a–c). Quantitation by real-time PCR revealed a nearly fourfold increase in mRNA level of *PUMA* in gastritis tissue compared with uninvolved tissues (Fig. S1A), which was further confirmed by western blotting (Fig. 1d). In addition, PUMA expression was found to be significantly correlated with the severity of gastritis, which was marked by elevated apoptosis compared with the uninvolved tissues using terminal deoxynucleotidyl transferase-mediated dUTP-fluorescein nick end labeling (TUNEL) staining (Figs. 1e, f and S1B). Western blot of caspase3 and cleaved-caspase3 and IHC analysis of cleaved-caspase3 also verified the correlation between PUMA expression and elevated apoptosis (Fig. S1C, D). In addition, western blot analysis of PUMA expression revealed increased induction of *PUMA* in most *H. pylori*-positive gastric cancer tissues compared with the negative controls (Fig. S1E). The above observations prompted us to further investigate the role of PUMA in gastritis using human cell lines and mouse models. Upon incubation with *H. pylori* at a ratio of 100:1 (bacteria to cell), we found that PUMA mRNA levels were increased by eightfold within 24 h in the human GEC line AGS (Fig. 1g). In addition, we used mice infected with *H. pylori* to determine PUMA expression. Western blot and IHC analysis were performed at 24 h, 48 h, and 7 days after *H. pylori* treatment. As shown in Figs. 1h and S1F, PUMA protein was induced after *H. pylori* treatment and elevated from 24 h, 48 h to 7 days. Collectively, we reasoned that PUMA might play roles in *H. pylori*-induced gastritis and gastric cancer, while *H. pylori* infection can lead to rapid induction of PUMA in GECs.

### PUMA induced apoptosis in an *H. pylori*-treated gastric cancer cell line

**Fig. 2:**
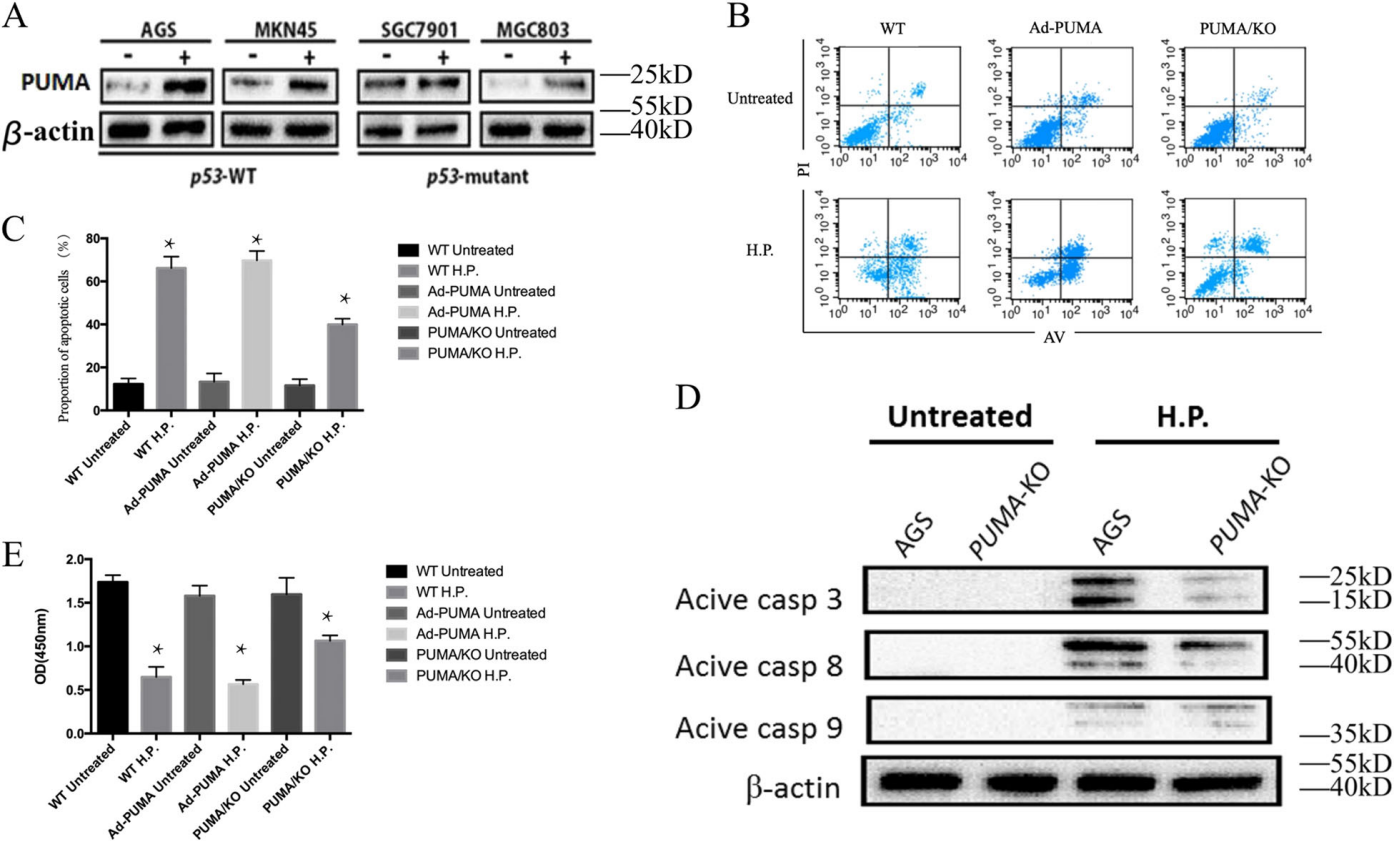
PUMA and apoptosis induction altered by *H. pylori* in gastric cancer cells. **a** The indicated gastric cancer cell lines with different p53 statuses were treated with *H. pylori* for 60 h. *PUMA* expression was analyzed by western blotting. **b**, **c** WT and *PUMA*-KO AGS cells were incubated alone or in the presence of *H. pylori* for 60 h, and apoptosis and proliferation were then examined by flow cytometry. The results were repeated for more than three times, and representative pictures are shown, *N* = 5/group; the results are presented as the mean ± SEM. *P < 0.05. **d** Western blot analysis of active caspase3, caspase8, and caspase9 in WT and *PUMA*-KO AGS cells with or without *H. pylori* treatment for 60 h. **e** AGS cells were transfected with either a control adenovirus or ad-*PUMA* and then treated with *H. pylori* for 60 h. Proliferation were then examined by the CCK assay, *P < 0.05. The results were repeated for more than three times, and representative pictures are shown.

PUMA is a downstream target of p53 and plays a critical role in mediating both p53-dependent and -independent apoptosis^20^. To probe the potential role of p53 in PUMA induction, gastric cancer cell lines with different p53 status were treated with *H. pylori* for 60 h. The expression of p53 in both p53-wild type (p53-WT) and -mutant cell lines was elevated after *H. pylori* treatment (Fig. S2A). While PUMA expression was also elevated in both p53-WT and -mutant cell lines, it suggested p53-independent activation of *PUMA* (Fig. 2a). We further generated *PUMA*-KO AGS cells using CRISPR to determine whether *PUMA* plays a role in apoptosis following *H. pylori* infection (Fig. S2B). The success of *PUMA* knockout in AGS cell lines was confirmed by DNA sequencing and western blot (Fig. S2C). Using flow cytometry, we found a significantly smaller proportion of apoptotic cells in *PUMA* knockout (KO) cells by 60 h (Fig. 2b, c), with increased cell viability, compared with the control groups. Furthermore, AGS cells were infected with a PUMA-adenovirus or control for 24 h and then treated with *H. pylori* for 60 h (Figs. 2b and S2D). The proportion of apoptotic cells in Ad-PUMA-infected cells was not significantly increased compared with the control groups (Fig. 2c). Western blot analysis indicated reduced activation of caspase3 and caspase8 in *PUMA*-KO AGS cells compared with WT cells. Consistent with the flow cytometric analysis, we observed a decreased cleaved-caspase3 and caspase8 in *PUMA*-KO AGS cells (Fig. 2d). In addition, we used CCK8 to detect apoptotic cells after *H. pylori* treatment and similar results were found (Fig. 2e). These results indicated that PUMA plays a critical role in cell apoptosis induced by *H. pylori*.

### *PUMA*-deficient mice are resistant to *H. pylori*-induced gastritis apoptosis

**Fig. 3:**
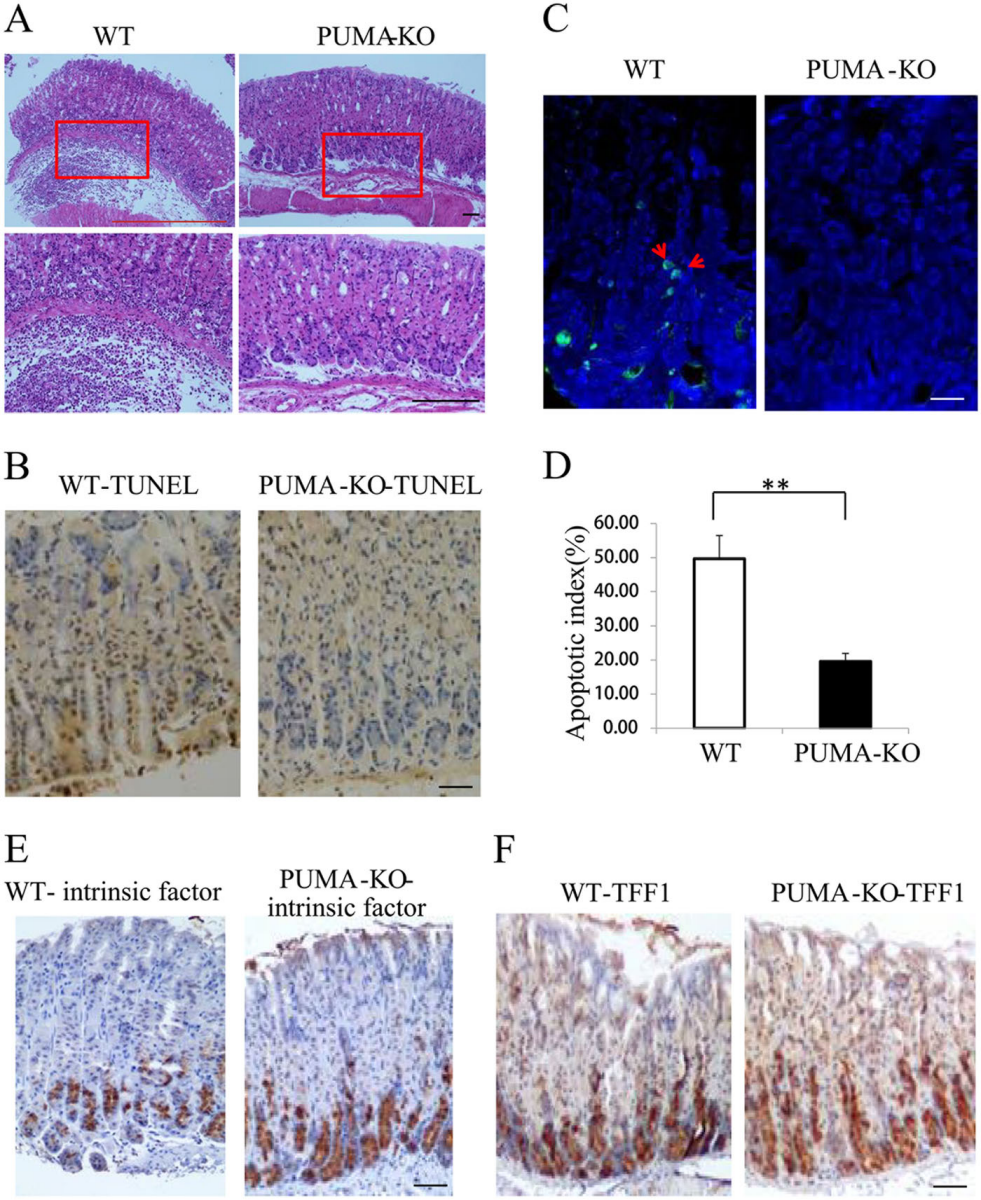
Suppression of *H. pylori* infection-mediated carcinogenesis, apoptosis, and differentiation in PUMA-deficient mice (WT and *PUMA*-KO mice were treated with *H*. *pylori* for 2 months to induce gastritis). **a** H&E staining of gastric tissues from WT and *PUMA*-KO mice after *H. pylori* treatment for 2 months (×100). Boxed regions of the glands are magnified in the lower panel (×200). **b** TUNEL IHC staining of gastric tissues from the treated mice (×200). **c** TUNEL (green) IF staining of gastric tissues from the treated mice after *H. pylori* treatment for 2 months, *N* = 5/group; the results are presented as the mean ± SEM. **d** The apoptosis index was measured by counting TUNEL signals in 100 randomly selected glands. **P < 0.01. **e** Staining for intrinsic factor showed mature zymogenic cells in *H. pylori*-treated wild type (left) and *PUMA*-KO (right) mice for 2 months. **f** Staining for TFF1 showed surface mucous cells in wild type (left) and *PUMA*-KO (right) mice treated with *H. pylori* for 2 months.

To determine whether the induction of *PUMA* contributes to *H. pylori*-induced chronic gastritis in vivo, we compared the pathological state and apoptosis using WT and *PUMA*-deficient (*PUMA*-KO) mice. Two months after infection, all mice were sacrificed and the infection status was verified by Giemsa staining and rapid urease test (Fig. S3A, B). Hematoxylin–eosin (H&E) staining revealed that submucosal inflammation was significantly blunted in *PUMA*-KO mice after *H. pylori* treatment for 2 months (Fig. 3a). Apoptosis was detected by TUNEL staining and western blot of caspase3 and cleaved-caspase3 as well as IHC analysis of cleaved-caspase3 in the gastric mucosa after *H. pylori* treatment for 2 months. Apoptosis markedly increased in WT mice, which was blocked in *PUMA-*KO mice (Figs. 3b–d and S3C, D). Interestingly, we also found that the expression of intrinsic factor, a glycoprotein that is essential for the absorption of cobalamin, and TFF1 (trefoil factor 1), which is mainly expressed in the fundus and antrum of gastric mucous cells^21^, increased in *PUMA*-KO mice (Figs. 3e, f). These results confirm the critical role of PUMA in GEC apoptosis and gastritis.

### p65 binds to the *PUMA* promoter to activate *PUMA* transcription following *H. pylori* treatment

**Fig. 4:**
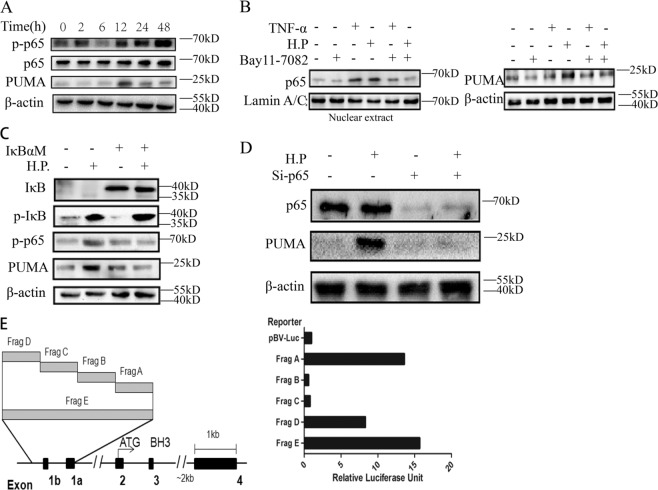
P65 directly binds to the *PUMA* promoter to activate its transcription following *H. pylori* treatment. **a** Expression of *p-p65* (*S536*), *p65*, and *PUMA* on AGS cells after treatment with *H. pylori* at the indicated time points was analyzed by western blotting. **b** AGS cells were treated with 10 mmol/L BAY 11–7082 for 1 h and then with *H. pylori* or 10 ng/mL TNF-α for 24 h. Left, nuclear fractions were isolated from cells and analyzed for *p65* expression by western blotting; right, western blot analysis of *PUMA* and *β-actin* expression in whole-cell lysates after *H. pylori* or TNF-α treatment. **c** WT AGS cells were transfected overnight with pCMV or IκBαM and then treated with *H. pylori* for 24 h. The expression levels of *PUMA,* *p-IκB*, *IκB*, and *p-p65* were analyzed by western blotting. **d** AGS cells were transfected with either a control scrambled siRNA or a p65 siRNA for 24 h and then treated with *H. pylori* for 60 h. *p65* and PUMA expression was probed by western blotting. **e** Left, schematic representation of the genomic structure of *PUMA* highlighting the *PUMA* promoter fragments (Frag) A–E of the *PUMA* promoter, followed by treatment with *H. pylori*. Reporter activities were measured 24 h later by a luciferase assay.

**Fig. 5:**
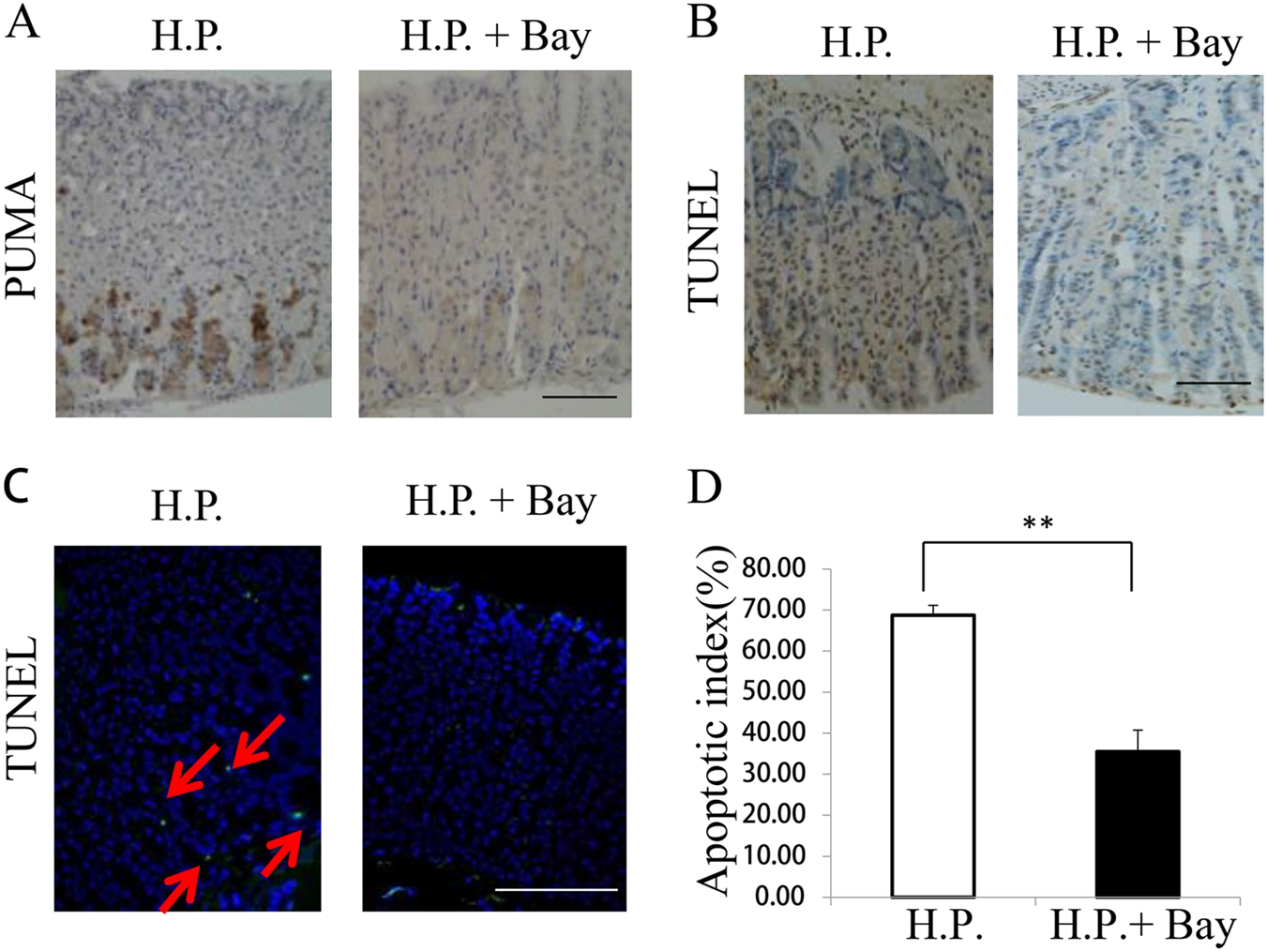
Effects of NF-κB inhibition on *H. pylori*-induced apoptosis and PUMA expression (WT mice were treated with *H*. *pylori* alone or in combination with 8 mg/kg of the NF- κB inhibitor Bay 117082 for 3 days). **a** IHC staining of PUMA in gastric tissues from the treated mice. **b** TUNEL (brown) staining of gastric tissues from the treated mice. **c** TUNEL (green) IF staining of gastric tissues from the treated mice. **d** The apoptotic index was calculated by counting TUNEL signals in 100 randomly selected glands following TUNEL staining, as in **c**. *N* = 4/group, the results are presented as the mean ± SEM, **P < 0.01 compared with the control.

We then explored whether p53-independent activation of *PUMA* is mediated by the canonical NF-κB pathway upon *H. pylori*-induced treatment. In the NF-κB signaling cascade, phosphorylation of p65 is required for nuclear translocation and transcriptional activation^22^. Thus we detected time-dependent increases in p-p65 (S536) and PUMA in AGS cells upon *H. pylori* treatment (Fig. 4a). Pretreatment with the NF-κB inhibitor BAY 11–7082 for 1 h blocked p65 activation (p-p65) and PUMA induction by *H. pylori* or TNF-α at 24 h (Fig. 4b). Transfecting cells with IκBαM, a nondegradable mutant of I κB, reduced *H. pylori*-induced PUMA expression and p65 phosphorylation in WT AGS cells (Fig. 4c), indicating that *H. pylori*-induced p65 activation is mediated by I κB depletion through the canonical NF-κB pathway. Knockdown of p65 using small interfering RNA (siRNA) completely inhibited PUMA induction at 60 h (Fig. 4d). Using reporter assays^19^, we found that fragments A, E (the proximal 495-bp region of the *PUMA* promoter), and D (the NF-κB responsive element distal region) were activated by p65 (Fig. 4e). Treatment of mice with the NF-κB inhibitor Bay11–7082 for 3 days (8 mg/kg/day) significantly inhibited PUMA induction (Fig. 5a) and apoptosis (Fig. 5b–d and S4A, B) in gastric tissues. These findings suggest that p65 directly activates *PUMA* transcription through binding to the distal site of *PUMA* promoter upon *H. pylori* treatment to induce GEC apoptosis.

### *H. pylori* treatment-induced activation of PUMA and gastritis is blocked by *TLR2*-KO

**Fig. 6:**
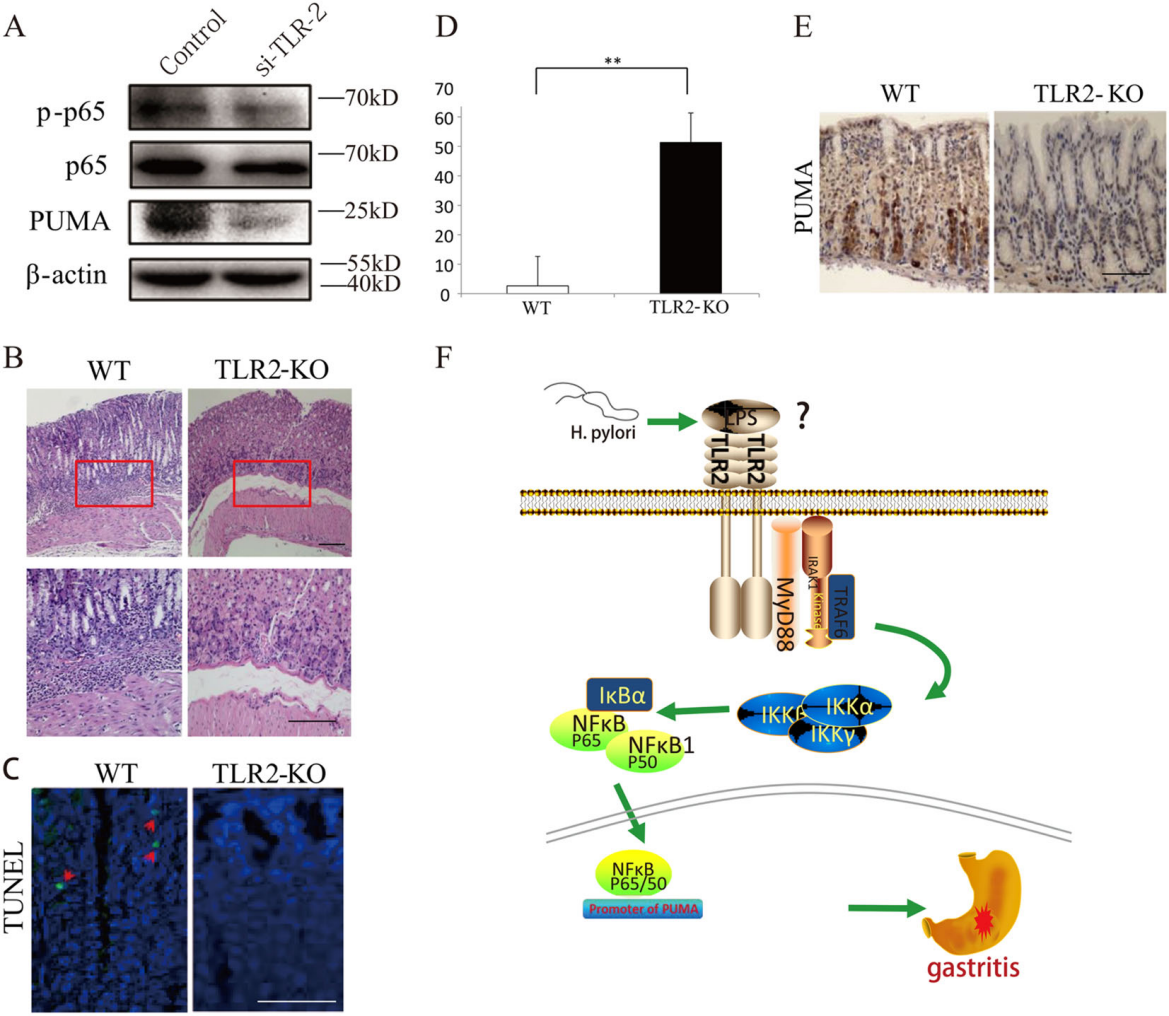
TLR 2 inhibition can suppress *H. pylori*-induced gastritis by downregulating *PUMA* (WT and *TLR2*-KO mice were treated with *H*. *pylori* for 2 months to induce gastritis, *N* = 5 for each group). **a** AGS cells were transfected with either a control scrambled siRNA or a TLR2 siRNA for 48 h and then treated with *H. pylori* for 24 h. *p-p65* and PUMA expression were probed by western blotting. **b** H&E staining of gastric tissues from WT and *TLR2*-KO mice after *H. pylori* treatment for 2 months (×100). Boxed regions of the glands were magnified in the lower panel (×200). **c**, **d** Representative images of TUNEL staining (green) of gastric tissues from treated mice. WT and *TLR2*-KO mice were treated with *H. pylori* for 48 h, *N* = 5/group; the results are presented as the mean ± SEM. **P < 0.01. **e** Representative images of PUMA IHC staining of gastric tissues from treated mice. WT and *TLR2*-KO mice were treated with *H. pylori* for 48 h. **f** Proposed roles of the TLR2 and PUMA involved in *H. pylori*-induced gastritis.

TLRs are pattern recognition receptors that are crucial for sensing pathogens, including *H. pylori*, and subsequent activation of the host immune response and NF-κB signaling^23^. We further investigated whether TLR2, which was previously implicated in the *H. pylori*-induced response, could mediate the activation of *p-p65* and *PUMA* in *H. pylori*-induced gastritis. Knockdown of *TLR2* by siRNA in AGS cells strongly decreased *H. pylori*-induced *p-p65* and *PUMA* expression (Fig. 6a). Furthermore, we used *TLR2*-KO mice to explore the role of TLR2 in *H. pylori*-induced gastritis. H&E staining revealed that submucosal inflammation was induced by *H. pylori* treatment in WT mice, but it was significantly blunted in *TLR2*-KO mice (Fig. 6b). TUNEL staining (green) revealed a tenfold decrease in GEC apoptosis in *TLR2*-KO compared with WT mice (Fig. 6c, d). The result was also verified by western blot analysis of caspase3 and cleaved-caspase3 and IHC staining of cleaved-caspase3 (Fig. S5A, B). IHC analysis further confirmed the reduced activation of *PUMA* in the *TLR2*-KO mice after *H. pylori* treatment (Fig. 6e). In addition, western blotting revealed that p-p65 and PUMA was induced by 2 months of *H. pylori* treatment in WT mice, but it was significantly blunted in *TLR2*-KO mice (Fig. S5C). These results suggest that TLRs likely play a key role in directly regulating GEC cell death during innate immune responses in acute inflammation.

## Discussion

The inflammatory response to *H. pylori* infection can initiate and accelerate a sequence of oncogenic events characterized by damage to the gastric epithelium, elevated apoptosis, and inappropriate cellular proliferation, which renders the cell vulnerable to further neoplastic changes^24^. Elevated apoptosis has been demonstrated to be associated with *H. pylori-*positive gastritis, while apoptotic cells are rare in the neck region of the gastric glands (the regenerative cell zone) in normal gastric mucosa. Following initial *H. pylori* infection, atrophic gastritis progresses, the regenerative cell zone shifts downward, and a relatively large number of apoptotic cells are observed^25^. However, the precise mechanisms of *H. pylori*-induced gastric cancer and GEC apoptosis have not been completely illustrated. In this study, we found that increased *PUMA* expression was correlated with the level of apoptosis, severity of human gastritis, and gastric cancer. *PUMA* deficiency in mice abrogated *H. pylori*-induced gastritis and GEC apoptosis, supporting the function of *PUMA* as a critical mediator of GEC apoptosis and a significant modulator of *H. pylori*-related gastritis. *PUMA* is necessary for TNF-α-induced apoptosis through intrinsic pathways^19^. Consistent with previous studies, we found that the activation of *PUMA* by NF-κB promoted caspase activation^26–28^.

Inflammatory conditions are characterized by activation of the transcription factor NF-κB, resulting in the expression of NF-κB-regulated, inflammation-related genes, such as inducible nitric oxide synthase and cyclo-oxygenase-2. In this study, we identified PUMA as a downstream target of NF-κB and a critical mediator of *H. pylori*-induced GEC apoptosis and gastric cancer. PUMA mRNA and protein were consistently activated in cells treated with both *H. pylori* and TNF-α. The induction of PUMA by *H. pylori* required the p65 subunit of NF-κB and was mediated by a IκB site located in the distal region of *PUMA* promoter. Our results showed that a deficiency in the p65 component of NF-κB, a key regulator of inflammation^17^, blunted PUMA induction in vitro and in vivo. This observation, along with the finding that NF-κB or TNF-α was activated in *H. pylori*-treated mice, suggested that NF-κB/TNF-α were responsible for PUMA induction and subsequent GEC apoptosis. These results established the direct regulation of a BH3-only Bcl-2 family member by NF-κB during acute gastritis via a p53-independent mechanism.

Several products are currently thought to be important for the activation of NF-κB by *H. pylori*: LPS, peptidoglycan, and CagA^29,30^. LPS has been shown to be recognized by host cells by binding to either TLR2 or TLR4 and has a role in *H. pylori*-induced NF-κB activation and the inflammatory response^23,31^. In macrophages, *H. pylori* activates NF-κB via TLR2 (for induction of interleukin (IL)-6 and IL-1β) and TLR4 (for induction of IL-12, IL-10, and IL-8)^23^. In our study, *p-p65* and *PUMA* were decreased after *TLR2* knockdown in the AGS cell line. In addition, H&E/IF staining revealed that the *TLR2*-KO mice were highly resistant to *H. pylori*-induced gastritis due to blocked NF-κB activation and PUMA induction. Our novel findings suggest that GEC death and PUMA induction are likely triggered by TLR2-mediated activation of NF-κB and amplified via subsequent induction of inflammatory cytokines. Further studies are needed to investigate which of these interconnected nodes in the signaling pathways might be pharmacologically modulated to improve epithelial healing and resolution of the immune response to prevent chronic gastritis and associated cancer.

In summary, our results demonstrate that TLR2/NF-κB-mediated PUMA induction contributes to the pathogenesis of *H. pylori-*induced gastritis by promoting GEC apoptosis. Inhibition of *PUMA* directly using a small molecular inhibitor^32^ or indirectly using anti-TLR2^33^ might represent a novel approach to reduce GEC death and to prevent the development of chronic gastritis.

## Materials and methods

### Tissue samples

The acquisition for all human tissues was approved by the Institutional Review Board of the Digestive Department of the First Affiliated Hospital of Nanjing Medical University. All subjects included 20 frozen pairs of matched *H. pylori*-positive gastritis and 20 uninvolved tissues recruited from the First Affiliated Hospital of Nanjing Medical University in 2017. *H. pylori* detection was confirmed by ^13^C breath test. Among the 20 patients in the gastritis group, gastritis tissue was found in the antrum of 6 patients, fundus of 3 patients, corpus of 7 patients, and cardia of 4 patients. Thirteen males and 7 females represented in the *H. pylori*-positive group, ranging in age from 37 to 69 years, whereas 14 males and 6 females comprised the uninvolved group, ranging in age from 24 to 73 years. Among the 20 patients in the gastritis group, none had received prior treatment. Cancer subjects included three *H. pylori*-positive gastric cancer and three negative gastric cancer tissues. All the sample studies have obtained patient consent and were collected by gastroscopy biopsy. Both of the tissues from human and mice were randomly selected and the investigators were blinded to the group allocation during the experiment.

### Cell culture and treatment

The gastric cancer cell lines were cultured in RPMI 1640 (Gibco) supplemented with 10% fetal bovine serum. All the cells were maintained at 37 °C with 5% CO_2_. The source and mycoplasma contamination of the cell lines were evaluated by Beijing YueWei Gene Technology Co., Ltd in October, 2013: DNA prepared from our cells using a commercial Chelex100 kit was analyzed by STR (Short tandem repeat) profiling. Cell lines were considered to be identical to the ATCC corresponding cell lines when the entered short tandem repeat (STR) profiles yield 100% match to the ATCC STR database. No cross-contaminated cell lines or mycoplasma contamination was detected. All the in vitro experiments were repeated three times. *H. pylori* bacteria was grown on Columbia agar plates (bioMérieux, Marcy) with selective supplement (Oxoid, Basingstoke, UK) under microaerophilic conditions using an anaerobic chamber (BBL Campy Pouch System, Becton Dickinson Microbiology Systems, San Diego, CA, USA) at 37 °C for 48–72 h. *H. pylori* bacteria utilized for all experiments is the cytotoxic (CagA+/VacA+) reference strain of *H. pylori* SS1 (ATCC)^34^. The densities of the bacteria were measured by the optical density (OD) at 660 nm [1 OD660 = 1 dens^8^ colony-forming units (CFU)/ml]. For *H. pylori* treatment, AGS cells were infected with the bacteria at a cell-to-bacterium ratio of 1:100 for the indicated times in the culture medium.

### Establishment of *PUMA* knockout cell line

To knock *PUMA* in AGS cell line, we cloned a single-guide RNA (sgPUMA-1: AAACGCGCACGCCAGGAGGGCAGC; sgPUMA-2: CACCGTAGAGGGCCTGGCCCGCGA) into the corresponding pCas9 vector. The constructed plasmid was sequenced and then transfected to AGS cells with Lipofectamine 2000. Later, we extracted the genomic DNA of transfected AGS cells and amplified the *PUMA* gene. Subsequently, we selected the single colony and confirmed the *PUMA* knockout AGS cell lines by DNA sequencing and western blot.

### Mice and treatment

The procedures for all animal experiments were approved by the Institutional Animal Care and Use Committee of the First Affiliated Hospital of Nanjing Medical University. Mice on the C57BL/6 background with different genotypes, including WT, *PUMA*-KO (PUMA−/−), and *TLR2*-KO (TLR2−/−), were generated by breeding and identified by PCR genotyping as previously described: The *PUMA* KO mice were originally from G. P. Zambetti and had been crossed to B6 backcrossed to the C57BL/6 background for >10 generations (F10), and the *TLR2*-KO mice were originally from Carsten J. Kirschning and had been crossed to 129SV backcrossed to the C57BL/6 background for >10 generations (F10)^35–37^. The mice were housed in microisolator cages and allowed access to water and chow ad libitum. Three-to-4-week-old littermates were treated with *H. pylori* by injection of 0.4 mL brucella broth containing 3 × 10^8^ CFU *H. pylori* using a feeding needle for 7 days to induce gastritis. Negative controls that were used in all experiments showed no significant submucosal inflammation after broth medium treatment for 2 months by H&E staining (Fig. S5D). Two months after infection, all mice were sacrificed, and the stomach tissues were collected for further analysis. For NF-κB inhibition, mice were injected 1 h prior to *H. pylori* treatment with 8 mg/kg of the NF-κB inhibitor BAY 117082 (EMD Biosciences) once daily and then sacrificed at the indicated time points.

### IHC and IF staining

The frozen tissues of gastritis cases and uninvolved tissues were used to prepare sections. Tissue sections (5 µm) were deparaffinized, rehydrated, and treated with 3% hydrogen peroxide, followed by antigen retrieval in boiling 0.1 M citrate (pH 6.0) buffer once for 10 min. The sections were then blocked with 20% rabbit serum for 30 min. PUMA staining was performed at 4 °C overnight using a rabbit anti-PUMA antibody (Prosci 3043) with Alexa 594 (Invitrogen) for signal detection. Cleaved-caspase3 staining was performed at 4 °C overnight using a rabbit anti-Caspase 3 antibody (Proteintech 19677–1-AP) for signal detection. Cells with positive staining were scored in at least 100 crypt sections and reported as the mean ± standard deviation (SD). Every scale indicates 100 µm.

### Analysis of mRNA and protein expression

Total RNA was extracted using TRIzol Reagent (Invitrogen) and reverse-transcribed using the High Capacity RNA-to-cDNA Kit (Takara). The expression of cDNA was quantified using Taqman Gene Expression Master Mix with an ABI 7900HT System (Applied Biosystems). The primer of *PUMA* is m-PUMA 3’-AGCAGCACTTAGAGTCGCC and PUMA 5’-CCTGGGTAAGGGGAGGAGT. Western blotting was performed using antibodies against human PUMA, murine PUMA (Abcam), p65 (Santa Cruz), active caspase3 (Cell Signal), phospho-p65 (Ser536) (Cell Signal), Bad (Cell Signaling Technology), and β-actin (Sigma), as previously described^14^. All the experiments were repeated three times.

### Analysis of tissue damage and histology

Histological analysis of gastric tissue was performed by H&E staining. Histological scores evaluating chronic inflammation were determined as 0–3 based on previously described criteria^38^: 0 = normal, 1 = mild, 2 = moderate, 3 = marked.

### Analysis of apoptosis by flow cytometry

The different treated cells were trypsinized and centrifuged at 12,000 × *g* for 5 min at 4 °C. The cells were washed in D-Hanks solution at 4 °C, and cell apoptosis was detected using the apoptosis kit (eBioscience, USA).

### Statistical analysis

For the in vitro and in vivo experiments, the data are presented as the mean ± SEM. Statistical analyses were performed using two-tailed Student’s *t* test for parametric data and Pearson’s chi-squared test (χ^2^) for categorical data. *P < 0.05 was considered to be statistically significant, and **P < 0.01 was considered to be statistically highly significant.

## Supplementary information

Supplementary Figure legends

Figure S1

Figure S2

Figure S3

Figure S4

Figure S5
