# Supplementary material for: PUMA-mediated epithelial cell apoptosis promotes Helicobacter pylori infection-mediated gastritis
Source: Cell Death Dis. 2020 Feb 20;11(2):139. doi: 10.1038/s41419-020-2339-x (PMC7033162; doi:10.1038/s41419-020-2339-x)
Supplement: Supplementary file 1 — Supplementary Figure legends [file 41419_2020_2339_MOESM1_ESM.docx]

**Supplementary Figure legends**

**Figure S1: Elevated *PUMA* expression and apoptosis in gastric mucosa following *H. pylori* treatment.**

(A) PUMA mRNA expression in uninvolved gastric and gastritis tissues were analyzed by real-time PCR. ** *P* < 0.01. (B) IHC staining of TUNEL in a patient with gastritis. Arrows (red) indicate example TUNEL-positive cells (400×). (C) Western blotting analysis of Caspase3 and cleaved-Caspase3 in uninvolved and gastritis tissues. (D) IHC staining of cleaved-Caspase3 in uninvolved and gastritis tissues (400×). (E) Western blotting analysis of *PUMA* in a patient with gastric cancers. (F) PUMA expression in WT mice treated with *H. pylori* for 1, 2 and 7 days was analyzed by IHC

**Figure S2: *p53* expression following** ***H. pylori* treatment and construction of *PUMA*-KO cell line.**

(A) Western blotting analysis of *p53* in both *p53*-WT and -mutant cell lines. (B-C) DNA sequencing and Western blotting analysis to confirm the success of *PUMA* knockout in AGS cell lines. (D) Western blotting analysis of PUMA expression in AGS cells infected with PUMA-OE-adenovirus.

**Figure S3: Verification of *H. pylori* infection and apoptosis in *PUMA*-KO mice.**

(A) Giemsa staining of *H. pylori* treated mouse gastric tissue. Arrows (black) indicate example *H. pylori* (400×). (B) Rapid urease test of *H. pylori* treated mouse gastric tissue. (C) Western blotting analysis of Caspase3 and cleaved-Caspase3 in control and *H. pylori* mouse gastric tissue. (D) IHC staining of cleaved-Caspase3 in WT and *PUMA-*KO mouse gastric tissue (400×).

**Figure S4: Elevated apoptosis in *H. pylori* infected mice treated with the NF-κB inhibitor Bay117082.**

(A) Western blotting analysis of Caspase3 and cleaved-Caspase3 in mouse gastric tissue treated with the NF-κB inhibitor Bay117082. (B) IHC staining of cleaved-Caspase3 in mouse gastric tissue treated with the NF-κB inhibitor Bay117082 (400×).

**Figure S5: Apoptosis and *p-p65* expression in *H. pylori* treated *TLR2*-KO mice.**

(A) Western blotting analysis of Caspase3 and cleaved-Caspase3 in WT and *TLR-2* KO mouse gastric tissue. (B) IHC staining of cleaved-Caspase3 in WT and *TLR-2* KO mouse gastric tissue (400×). (C) Western blotting revealed the expression of *p-p65* and *PUMA* after 2 months *H. pylori* treatment in WT and *TLR2*-KO mice. (D) H&E staining to show submucosal inflammation after broth medium treatment of two months for negative controls used in all experiments (400×).
